# Experiences of HIV–Related Stigma and Mental Illness Among HIV–Associated Meningitis Patients in Rural Uganda

**DOI:** 10.1002/brb3.71233

**Published:** 2026-01-29

**Authors:** Abigail Link, Sarah Iribarren, Paul Bohjanen, Mark Okwir, David Meya, Betty Nabongo, Danuta Kasprzyk

**Affiliations:** ^1^ Division of Infectious Diseases University of Rochester Rochester New York USA; ^2^ Department of Child, Family, Population Health Nursing, School of Nursing University of Washington Seattle Washington USA; ^3^ Department of Medicine, Division of Infectious Diseases and International Medicine, Department of Medicine University of Minnesota Minneapolis Minnesota USA; ^4^ Department of Public Health Sciences University of Rochester Medical Center Rochester New York USA; ^5^ Department of Internal Medicine, Faculty of Medicine Lira University Lira Uganda; ^6^ Infectious Diseases Institute, College of Health Sciences Makerere University Kampala Uganda; ^7^ Department of Medicine Lira Regional Referral Hospital Lira Uganda

## Abstract

**Background:**

Inflammation in and around the brain in patients with meningitis can lead to confusion, cognitive dysfunction, and behavioral changes associated with mental health disorders. Stigma associated with HIV or mental illness can complicate meningitis, leading to misdiagnosis or delays in diagnosis and care. The frequency of misdiagnosis and/or co‐occurrence of meningitis and mental illness among people living with HIV (PLWH) remains uncertain. We explored the experiences of meningitis patients and the barriers and facilitators to care related to HIV stigma and mental illness.

**Methods:**

We conducted a convergent mixed‐methods study to evaluate experiences of patients who were hospitalized with HIV‐associated meningitis from February 2017 to May 2022 at Lira Regional Referral Hospital in Uganda. Experiences among patients who survived and family members of patients who died were explored. Surveys were conducted to obtain demographic information, investigate stigma, and assess symptoms of mental illness. Semi‐structured interviews probed the overall experience of patients with HIV and meningitis regarding social support, mental health, and stigma.

**Results:**

Twenty‐four patients with HIV‐associated meningitis and 20 family members of deceased meningitis patients were enrolled. Family members reported that 80% of deceased patients experienced stigma, whereas 29.2% of surviving patients reported experiencing stigma. Combined responses from surviving patients and family members identified 31.8% of patients with mental illness symptoms described as overthinking, depression, and/or anxiety, while 60.8% experienced HIV‐related stigma. Among participants who died, family members reported mental illness symptoms in 40%, compared to self‐reports of 25% in survivors. Barriers to HIV care included (a) lack of HIV education, (b) mental illness symptoms, (c) lack of social support, and (d) stigma or shame. While common facilitators were (a) access to HIV clinics and ART medication, (b) having a life purpose, and (c) social support.

**Conclusion:**

Stigma and symptoms of mental illness were common among patients with HIV and meningitis, which likely affected antiretroviral therapy (ART) adherence and HIV care. Recommendations include (1) adding mental health screening during hospital admission for all meningitis patients, especially among PLWH, for early evaluation and treatment and (2) increasing community awareness to dispel misconceptions and reduce HIV‐related stigma.

## Graphical abstract

Meningitis patients living with HIV often struggle with medication adherence and seeking HIV care due to the lack of education, support, and HIV‐related stigma, which can contribute to the development of mental health conditions in some individuals.

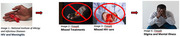

## 1 Introduction

The global estimates of meningitis infection affects over 2.5 million people annually (Wunrow et al. 2023), with the highest prevalence located within the meningitis belt stretching from Senegal to Somalia (World Health Organization [WHO] 2016). Meningitis represents inflammation of the tissues covering the brain, usually due to infection (World Health Organization [WHO] 2015), with the most common infectious forms caused by bacteria, fungi, viruses, and parasites (World Health Organization [WHO] 2020). Although meningitis can infect anyone, people at greater risk are children, the elderly, and those with impaired immunity, such as people living with HIV (PLWH) (World Health Organisation [WHO] 2023). Additionally, certain causes of meningitis are more common in PLWH, such as cryptococcal meningitis (CM) and tuberculosis meningitis (TBM) (Rajasingham et al. 2019) (Wilkinson et al. 2017).

Meningitis survivors often experience increased psychiatric disabilities (Johansson Kostenniemi et al. 2021). However, several case studies have called attention to circumstances whereby meningitis was misdiagnosed as psychosis or other mental illnesses due to similarities in clinical presentation (Sandler 1975; Rahim and Ghazali 2016; M. Kumar et al. 2024; A. Kumar et al. 2011; Thienhaus and Khosla 1984). This misdiagnosis can occur due to the inflammation around the brain, altering cognition and mental function (World Health Organization [WHO] 2020). These changes in cognition and behavior can also lead to alternative but misleading diagnoses such as psychosis or other mental health disorders, sometimes causing the diagnosis of meningitis to be missed or delayed.

PLWH are at higher risk of developing mental health disorders than individuals without HIV (US Department of Health and Human Services 2019), and HIV‐related stigma can exacerbate mental illness symptoms (Breet et al. 2014; Ruffell 2017; Akena et al. 2012). In Lira District, Uganda, recent surveys report the area has the second‐highest prevalence of HIV in the country at 7.5%. (Uganda Ministry of Health 2022) The global prevalence of depression among this population ranges from 22% to 44%, with higher prevalence among low‐ and middle‐income countries. (Rezaei et al. 2019) In Uganda, the prevalence for serious MH conditions among PLWH is 11.1%–17.4% (Akena et al. 2012; Lundberg et al. 2013) and is higher in certain parts of the country, such as northern Uganda, which faced two decades of rebel occupation by the Lord's Resistance Army (LRA). (Titeca and Costeur 2014) This conflict resulted in increased rates of post‐traumatic stress disorder (11.8%–54.0%) and depression (24.7%–67.0%) (Mugisha et al. 2015; Roberts et al. 2008).

**FIGURE 1:**
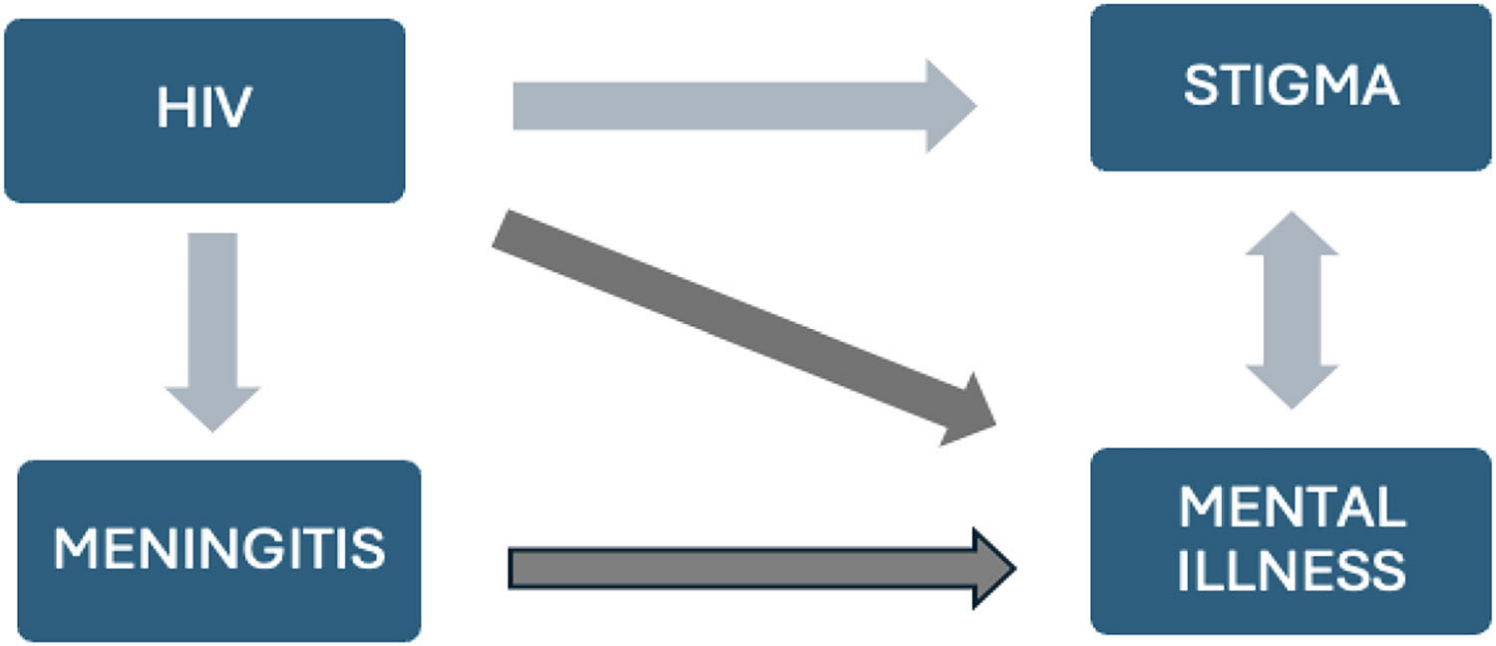
Relationships between HIV, meningitis, stigma, and mental illness.

Additionally, those with advanced HIV infection can develop HIV‐associated psychosis due to severe immunosuppression (Ursoiu et al. 2018). Despite evidence from case reports suggesting that mental illness occurs in patients with meningitis, the burden of mental illness among PLWH with overt meningitis is not well understood (A. Kumar et al. 2011; Thienhaus and Khosla 1984; Goeb et al. 2007). Although the current Uganda Clinical Guidelines for meningitis treatment include specific laboratory tests, treatments, and follow‐up (Uganda Ministry of Health 2023), these guidelines are often not followed in rural areas of Uganda, such as Lira Regional Referral Hospital (LRRH), our study site in northern Uganda, due to lack of expertise, infrastructure, and resources. Also, the current guidelines do not include recommendations for screening, diagnoses, or treatment of mental illness in patients with meningitis. In this study we sought to explore the experiences of patients and family members related to stigma and mental illness to better understand challenges to following current guidelines and to identify gaps in care that may lead to poor outcomes (Figure 1). Our goal was to explore the experiences of PLWH who were diagnosed with HIV‐associated meningitis, defined as meningitis of any etiology occurring in individuals with HIV, focusing on the barriers and facilitators to HIV and meningitis care related to stigma and/or underlying mental illness.

## 2 Methods

### 2.1 Study Design

This convergent mixed‐methods study evaluated patients with HIV‐associated meningitis who survived or family members who were caregivers of deceased patients hospitalized with meningitis from February 2017 to May 2022 at LRRH in rural northern Uganda. Quantitative and qualitative data were collected and analyzed sequentially to provide a comprehensive understanding of the patient's experience with meningitis, HIV care, stigma, and mental illness. Quantitative data were collected utilizing surveys, while qualitative data were collected through semi‐structured interviews. Each dataset was analyzed separately and merged to assess and identify areas of convergence or divergence in data results. This approach allowed us to integrate quantitative measures with the insights that qualitative methods provide for a richer understanding of the study objectives.

### 2.2 Setting

This study was conducted at LRRH, a government hospital located in rural, northern Uganda, with previous roots of rebellion occupied by the LRA from 1987 to 2006 (Titeca and Costeur 2014). During this occupation, the area was often cut off from the rest of the country, and the resources for healthcare were scarce. Currently, LRRH admits 1300 patients each month from nine surrounding districts. The hospital provides specialty services in pediatrics, surgery, ophthalmology, psychiatry, maternity, and palliative care (World Health Organization [WHO] 2017).

### 2.3 Sample Population

Our sample population was selected from an existing meningitis study that previously enrolled patients with suspected meningitis from February 2017 to May 2022 at LRRH. Eligible participants enrolled in our study included patients ≥13 years of age, living with HIV, diagnosed with laboratory‐confirmed CM or bacterial meningitis (BM), who were treated at LRRH, and for whom contact information was available. For patients who died, adult family members (age ≥18 years) who were caregivers during the patient's hospitalization were also enrolled. Due to the high mortality of HIV‐associated meningitis, family members were used as proxies to assess and understand the experiences of patients who died.

### 2.4 Procedures

Participants were purposively sampled by locating contact details through clinical records and were invited to participate by telephone or during follow‐up appointments. Separate sampling was conducted between those who died and those who survived to maintain a similar sample distribution. Study recruitment was conducted from February 10 to March 15, 2020, and August 12, 2021 to June 30, 2022 (recruitment interruption due to Covid‐19). Twenty‐four participants who survived and 20 adult family members/caregivers of deceased patients were enrolled. Data saturation from the semi‐structured interviews was reached after completing interviews with the 44 participants.

Surveys were conducted first, immediately followed by semi‐structured interviews of all consented participants. The survey instruments included demographic questions, HIV health‐seeking information, a stigma assessment with questions obtained from the 2016 Uganda Demographic Household Survey (UDHS) and 2011 Uganda AIDS Indicator Survey (UAIS), and items assessing knowledge about meningitis. Surveys and interviews were conducted in English or Leb Lango by trained interviewers fluent in both languages in a private office located within the grounds of the hospital. Surveys were completed by the interviewers using the REDCap survey platform, including verbal confirmation by participants. The surveys lasted approximately 20–25 min, while semi‐structured interviews lasted between 30 and 45 min.

The interviews explored patient's experiences with meningitis, HIV care, stigma, mental illness, social support, antiretroviral therapy (ART) use, and health‐seeking behaviors. Interviews with family members assessed perceptions regarding the experiences of deceased patients. Qualitative data from interviews were transcribed in the original language. Transcripts in Lango were translated into English, and 20% were periodically checked for consistency and accuracy by the study PI and interviewers.

### 2.5 Theoretical Model

**FIGURE 2:**
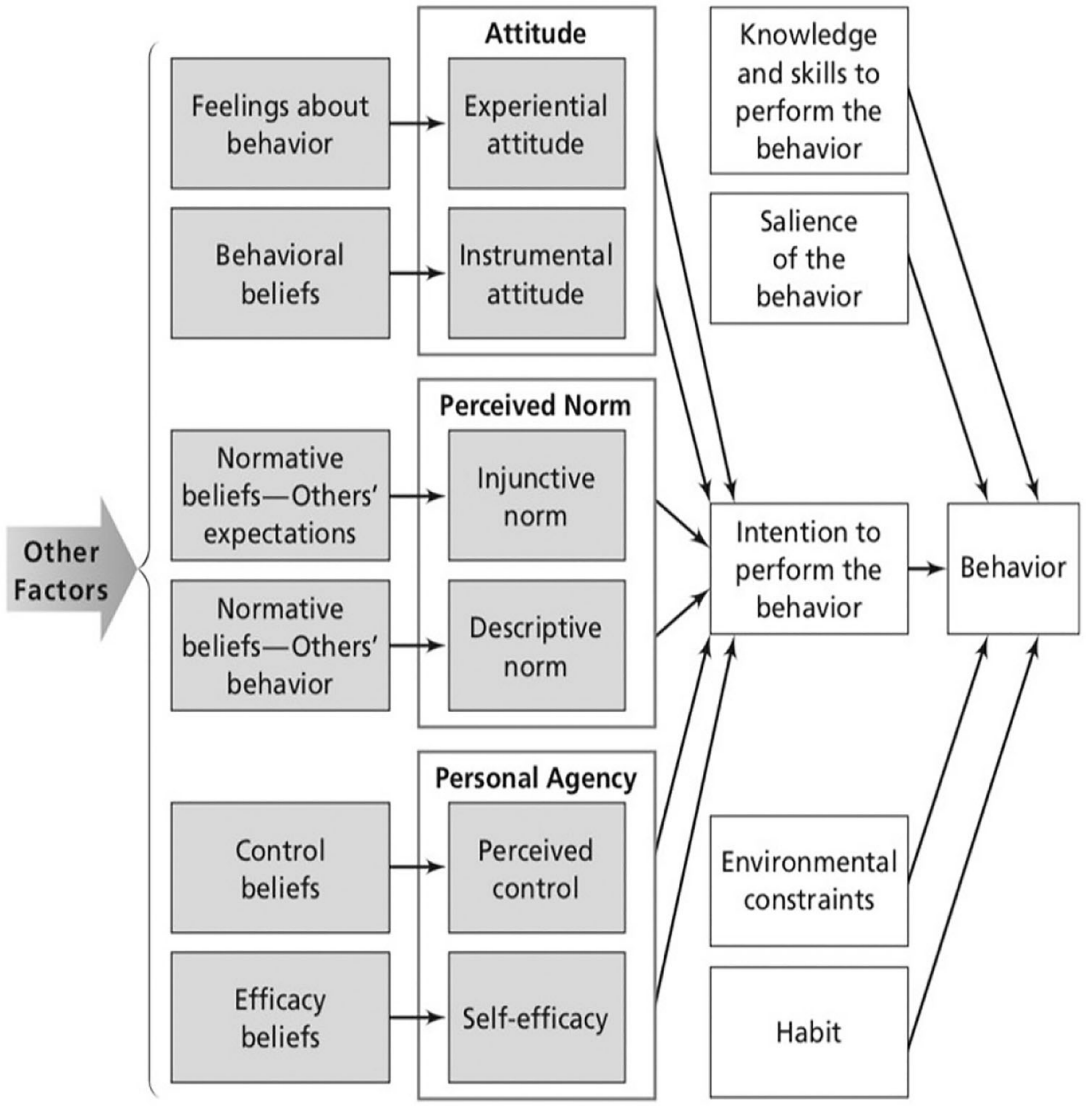
Integrated behavioral model.

The study was guided by the Integrated Behavioral Model (IBM), an extension of the Theory of Reasoned Action and the Theory of Planned Behavior (Montaño and Kasprzyk 2015). This model incorporates key constructs of attitude, perceived norms, and personal agency, and includes factors such as the environmental constraints, habit, knowledge, and salience of behavior (Figure 2). The IBM framework informed the design of survey and semi‐structured interview questions to identify the barriers and facilitators for behaviors related to meningitis and HIV care and treatment. It also guided the analyses of these determinants, which are hypothesized by IBM. Incorporating this model in the studies provided greater understanding of the gaps and drivers around meningitis and HIV care as well as experiences with these conditions and which key constructs motivate health behavior.

### 2.6 Study Variables

The dependent variable for this study was patient health behaviors. The IBM model was used to organize the key independent factors and constructs that influence these behaviors. The independent variables we assessed were patient attitudes, perceived norms, personal agency, environmental constraints, habits, knowledge, and intention. These variables were used to describe the conceptual relationships between patients with underlying HIV and meningitis diagnoses and their health behaviors. The purpose of these variables is conceptual and exploratory; they were not intended to produce statistical inferences or generalizability.

### 2.7 Analysis

Quantitative survey data were analyzed using descriptive statistics, and comparisons between groups were conducted with Fisher's Exact Test using R version 4.4.1. (R Core Team 2020) Qualitative data were analyzed inductively and deductively. (Bingham 2023; Elo and Kyngäs 2008) Inductive content analysis involved open coding and memo writing to capture any themes that emerged from the data without the influence from the IBM framework. The emergent themes were then compared and mapped back to the IBM framework to assess fit, highlight gaps, and identify potential extensions. In addition, deductive analysis was then guided by the constructs of the IBM framework, which were used as predefined categories to guide coding and interpretation, allowing us to identify how participant responses aligned with established constructs such as norms, facilitators, constraints, and attitudes. ATLAS.ti 9 was used to organize the interviews to build codes, networks, and memos to identify the major themes related to patient emotions and behavior toward ART, HIV diagnosis, stigma, and mental illness symptoms. All transcripts were reviewed for accuracy, validity, and consistency in coding. Triangulation of qualitative and quantitative data were explored to assess convergent and divergent findings.

## 3 Results

### 3.1 Participant Demographics

Among the 44 patients evaluated, 20 of the patients who died had CM, and among those who survived, 20 had CM and 4 had BM. The average age of patients was 37.6 years (SD: 12.5), with the majority being male (59%). Most patients were employed (*n* = 24, 54.5%) with a primary‐school level of education (*n* = 24, 54.5%). (Table 1) All patients were living with HIV, and most started ART prior to their meningitis diagnosis. Of those who died, 90% (*n* = 18) had previously started ART, while 83.3% (*n* = 20) of those who survived had started ART (Table 2). Among deceased participants, 45% (*n* = 9) stopped ART prior to their meningitis diagnosis, while 29.2% (*n* = 7) of those who survived stopped ART. For those who stopped ART, the reasons for stopping were related to lack of support to continue, medication side effects, and pill fatigue. However, 95.8% (*n* = 23) of those who survived their hospital stay restarted ART by the time of their discharge, while 70% (*n* = 14) of the patients who died were on or restarted ART at the time of death.

**TABLE 1:** Study participant demographics.

	Expired patient	Surviving patient	Total
	*n* = 20	*n* = 24	*n* = 44
**Age** (mean, SD)	34.9, 12.0	40.2, 12.5	37.6, 12.5
	***n*, %**	***n*, %**	*n*, %
**Sex**: Female	10, 50.0	8, 33.3	18, 40.9
**Marital status**: Married	10, 50.0	12, 50	22, 50.0
Employment status			
Full time	7, 35.0	8, 33.3	15, 34.1
Part‐time	0	4, 16.7	4, 9.0
Seasonal	3, 15.0	2, 8.3	5, 11.4
Unemployed	10, 50.0	10, 41.7	20, 45.5
Education			
Post‐secondary	3, 15.0	4, 33.3	7, 15.9
Secondary	4, 20.0	5, 20.8	9, 20.5
Primary	12, 60.0	12, 50.0	24, 54.5
None	1, 5.0	1, 4.2	2, 4.5
Meningitis etiology			
CM	20	20	40, 90.9
BM	0	4	4, 9.1

**TABLE 2:** ART experience results.

	Expired patient	Surviving patient	*p*‐value
	*n*, %	*n*, %	
ART started			
Yes	18/20, 90%	20/24, 83.3	0.52
ART stopped			
Yes	9/20, 45.0	7/24, 29.2	0.27
Reason for stopping ART			
Thought they were healed	2/9, 22.2	0	NA
Unsupportive environment	2/9, 22.2	3/7, 42.9	0.38
Pill fatigue	3/9, 33.3	0	NA
Side effects	1/9, 11.1	3/7, 42.9	0.15

### 3.2 Interviews

Surviving patients or family members of deceased patients reported several barriers and facilitators of HIV care. Key barriers included lack of HIV education, mental illness symptoms, lack of support, stigma, and shame. Key facilitators included access to HIV clinics and ART medication, habit, support, and purpose in life. A list of codes, definitions, and the occurrence frequencies are found in Table 3.

**TABLE 3:** Interview code results.

Codes	IBM construct factors	Definitions	Deceased patient *n*, %	Surviving patient *n*, %
Barriers to HIV care				
Lack of HIV education	Education	Limited HIV education by healthcare providers about HIV topics such as cause, symptoms, prevention, diagnosis, treatments, and side effects	3, 15.0	6, 25.0
Lack of support	Perceived norms	Lacked encouragement from friends, family, or healthcare staff	13, 65.0	9, 37.5
Mental illness symptoms	Perceived norms	Acknowledged mental health issues of depression, anxiety, or overthinking	8, 40.0	6, 25.0
Stigma/shame	Attitude perceived norms	Experienced stigma related to HIV diagnosis and/or associated feelings of shame	16, 80.0	7, 29.2
Facilitators to HIV care				
Access to HIV clinics and ART	Environmental constraints	Acknowledged facilitators of physical access to or available transport to HIV clinics and available ART medication	18, 90.0	23, 95.8
Purpose	Attitude	Experienced feelings of purpose or having something or someone to live for	4, 20.0	5, 20.8
Support	Perceived norms	Acknowledged encouragement and support	20, 100.0	23, 95.8

### 3.3 Barriers to HIV Care

#### 3.3.1 Lack of Education Diminished Participants’ Understanding of HIV

All participants (surviving patients and family members) reported some education related to HIV, but gaps in education were identified, as some reported they did not receive education on some HIV topics, such as ART.

…no one told me about it [ART]… they just give you the medicine and then you go and start using it with your own knowledge. (Patient, male, age 34)

…even by that time, (pause) I just come and get my drugs, but I don't know why I am getting it. Then with time, I kept coming then, the way I realized it, then I always read what is written on the walls. (Patient, male, age 24)

Some participants also described delays in starting ART treatment for years, citing the absence of symptoms and unnecessary medication.

I was not sick. There was no knowledge. I was not sick at all. OK, after I became sick, and they confirmed it, I was like, oh, that was real. (after testing positive three‐years earlier; Patient, male, age 48)

#### 3.3.2 Shame, Fear, and Bullying Contributed to HIV Stigma

Among those who experienced stigma, three key challenges were faced. The most frequent were feelings of shame, fear, and being bullied. These feelings came from many sources, but stigma was reported as a major contributor. The following responses from participants reflect how shame and fear directly impacted their lives and ability to seek HIV treatment:

##### 3.3.2.1 Shame Prevented Patients From Taking ART

He would feel a lot of shame that people would laugh at him, that this person is on ARV (antiretrovirals). That[‘s] what people in the village fear… (Family member of 60‐year‐old male patient)

If you find receiving ART difficult, it means you are afraid; you are ashamed of yourself, and you have the fear that people will talk about your condition. So, because [of] this, you will find it very difficult, and you will not receive you[r] drugs. (Patient, male, age 40)

##### 3.3.2.2 Fear of Collecting ART Medications

The fear of having their HIV status exposed paralyzed some participants from collecting, receiving or taking their ART.

…she would fear that people would laugh at her, and people would talk that this person is sick, [and] saw him/her getting the drugs. That is what brings fear. (Family member of 48‐year‐old female patient)

I think it could be fear because whatever brings shame start(s) with fear… but for him, he was fearing. (Family member of 37‐year‐old male patient)

##### 3.3.2.3 Bullying by Community Members

Another common source of stigma was bullying or making fun of people, based solely on their HIV status.

…she fears because she would say that people laugh at people if you have the HIV virus. They laugh at people, and they like bullying them. They like bullying people. (Family member of 32‐year‐old female patient)

Sometimes, they can just say that ‘You people who are having HIV, you're just wasting your time. You can die any, any day and any time. You're not supposed to even stay with us because you are now hopeless. (Patient, female, age 33)

### 3.4 Mental Illness Symptoms Were Common Among Those Who Experienced HIV Stigma

Participants who experienced HIV stigma (a perceived attitude or a perceived norm) more frequently reported mental illness symptoms such as overthinking and anxiety, as the majority of those who experienced HIV stigma reported symptoms of mental illness. Terms such as “depression” and “anxiety” are not commonly used in this region; however, idioms of distress such as “thinking too much” or “overthinking” were more frequently expressed (Chibanda et al. 2016; Patel et al. 1997; Haas et al. 2021).

I think of a lot of things like how my life is because, I am positive. That is why I never stop having bad thoughts. (Patient, female, age 32)

I think he used to think people with HIV die at any time, so that was only his worry, so, he would say I should not over torture myself taking these HIV treatment and then I die, so it's better I don't take and die. (Family member of 26‐year‐old male patient)

### 3.5 Mental Illness Symptoms Affected ART Adherence

For those who had experiences with depression or anxiety, it was commonly manifested by the inability to take daily medications. Feelings of depression demotivated participants, and some stopped their medications for HIV treatment.

Because if I get depressed, I find it hard to take my HIV meds.(Patient, female, age 33)

### 3.6 HIV Stigma Affected More Participants Who Died Compared to Those Who Survived

HIV‐related stigma remains prevalent in Uganda, as many participants reported their experiences with it. Additionally, stigma was reported more frequently by family members of those who died compared to reports from those who survived.

…she had that stigma which was killing her sincerely! (Family member of 40‐year‐old female patient)

…big people like her parents even said some things that would break her heart. They didn't give her what she wanted, and this created even more stigma. Then she became so angry, and she just thought that it is better to die. (Family member of 35‐year‐old female patient)

### 3.7 Symptoms of Mental Illness Were Reported by Family Members of Those Who Died

Among patients who died, their family members reported symptoms of mental illness. Family members shared the patient's statements and experiences related to thoughts of hopelessness, overthinking, and mortality. These data do not aim to establish associations between mental illness and death. Instead, it highlights the sentiments family members shared related to their perception and experience around their loved one's behaviors, death, and mental state.

The reason why she got hard to take ARV, (pause) is because she was so angry, hmmm. Because she was seeing that no one cared about her, (pause) which made her to believe that she can't get well, and that made her to just think of dying… (Family member of 35‐year‐old female patient)

She would agree to take the drugs, only that it was the fun makers [people making fun of her] that made her to overthink, and she died. (Family member of 48‐year‐old female patient)

### 3.8 Facilitators to HIV Care

There were also several facilitators that participants reported, which aided in their acceptance of HIV and meningitis care and treatment. These key facilitators included access to ART, maintaining habits for medication adherence, social support, and purpose in life.

### 3.9 Access to ART Enables Adherence

ART availability and access to health facilities were key factors in the accessibility of quality healthcare and supported ART adherence. From the participants’ reflections, access to health care affected multiple aspects of their lives, including their ability to receive ART and obtain HIV care. Access was defined as a construct of environmental constraint based on the IBM theoretical model.

Access to an ART clinic is a major facilitator for ART adherence. The experience at the ART clinics was also important for most participants. Of particular importance was timely ART distribution, decreased pill burden, and living near a clinic or having easy access to transportation to a clinic. These were all facilitators for receiving ART and important factors for ART adherence, as conveyed by the following quotes:

I came and I was given the [HIV] drugs and went back with it. It was easy because the drug was available. If not, it was going to be difficult. (Patient, female, age 50)

Yeah, the distance from the… hospital was not that far. (Family member of 29‐year‐old female patient)

### 3.10 Family and Healthcare Provider Support Provides Encouragement and Purpose

Most participants felt that they had some support or were a source of support for their family member during their experience with meningitis and HIV care. Those who received support reported positive experiences with HIV care and felt encouraged. In contrast, those who did not have support reported more self‐isolation and symptoms of depression and anxiety. Having positive experiences at the ART clinic through relationship building with health workers, other clients, and other sources of support helped facilitate consistent HIV care for patients.

Healthcare staff said] that I should not fear taking the medicine, since they were giving me the medicine. If I stopped taking it, I would die. They told me with a good heart and not with harassment. Then I started using the medicine by that time very well… (Patient, male, age 35)

Additionally, visits from friends and family provided encouragement and gave them a sense of belonging and renewed focus for life, as demonstrated by these comments:

Some of his relatives would visit him and his friends. So, I just tell them, if the patient is there, it's not good *not* to visit, so people were visiting him. They could encourage him. (Family member of 29‐year‐old male patient)

I got it easy because I kept seeing from my friends. So, I just decided that if you are taking the drugs, that is what makes you healthy. You don't get any problem so long as you[’re] adhering well to your drugs as instructed…I'm just taking it that way, and that's why I'm still alive till now. (Patient, female, age 42)

## 4 Discussion

Our study findings demonstrated that PLWH and meningitis often experience HIV‐related stigma, which affects their ability to take ART and other medications. Stigma contributed to symptoms of mental illness and feelings of fear and shame. Failure to take ART leads to immunosuppression, which increases the risk of developing meningitis. However, not all patients experienced stigma; some were supported and encouraged by friends, family, and healthcare workers. To our knowledge, this study is the first to explore HIV and mental illness experiences among meningitis patients and their family members. Centering on the experiences of patients and family members provided unique insights and revealed key themes, which affected their perceptions around HIV education, mental health, stigma, and support.

Stigma continues to be a key factor contributing to lack of ART adherence ever since HIV medications became available (Sayles et al. 2009). We found that stigma still plays a profound role in people's lives, directly related to their HIV status. Stigma not only influences the ability to take life‐saving drugs, but it also plays a significant role in the ability to live life and have a sense of purpose. More patients who died were impacted by or had previous experiences with stigma, compared to those who were still alive. Although perceptions of family members may not reflect the actual experiences of those who died, it is remarkable that 80% of family members of those who died reported their deceased family member experienced stigma. No studies have found a direct causal link between stigma and mortality, although stigma is a moderator, which affects the ability or desire to get tested for HIV (World Health Organisation [WHO] 2011; England Public Health. 2019 ), adhere to ART (Sayles et al. 2009), and engage in HIV care (World Health Organisation [WHO] 2011). Many patients or family members who perceived stigma had feelings of fear and shame associated with their HIV status, which was exacerbated during interactions with their community, family, friends, and healthcare workers.

According to the findings in the UAIS, “stigma leads to secrecy and denial that hinder people from seeking counselling and testing for HIV, as well as care and support services.” (Uganda Ministry of Health (MOH) 2012). The participants’ experiences supported this assertion, as they reported that fear and shame due to stigma did contribute negatively to their decisions around seeking treatment for HIV and adherence to medication. The effects of stigma continue to be a major barrier to HIV care and likely play a role in opportunistic infection acquisition, specifically CM.

Mental illnesses such as depression and anxiety are often neglected in HIV care and are commonly misunderstood in Uganda (Mugisha et al. 2019). Screening and treatments for mental illness are often unavailable due to limited access to mental health services (Sessions et al. 2017; Molodynski et al. 2017). Mental illness is still an unrecognized condition for many Ugandans (Mutegeki 2019; Shumba et al. 2013), despite serious mental illness being prevalent among PLWH, with rates ranging from 11.05% to 17.4% (Akena et al. 2012; Lundberg et al. 2013). Our participants acknowledged that depression played a role in the ability to seek HIV and meningitis care, while stigma and lack of social support contributed to mental illness symptoms.

A study in Uganda found that 7.9% of participants with depression also experienced AIDS‐related stigma (Akena et al. 2012). We found that 60% of our participants who experienced HIV stigma reported mental illness symptoms. Formal diagnosis of mental illness in this rural hospital setting is rare, as only one participant had a formal diagnosis of depression at the time of our study. A meta‐analysis exploring the association of HIV‐related stigma and health outcomes found that stigma was associated with lower social support and increased anxiety and mental distress (Rueda et al. 2016), which was also reported by our participants. We also found that those who had more support from family and friends reported fewer experiences with depression and anxiety, compared to those who had less support or had strained relationships, a finding also supported by a previous study conducted in Uganda (Rueda et al. 2016). Limited research has been conducted on mental illnesses related to meningitis among PLWH, yet mental illness appears to play a role in the relationship between ART adherence, overall health, and health‐seeking behaviors. The presence of mental illness is important to understand when assessing someone's ability to maintain their health in order to adhere to the prolonged regimens needed for successful meningitis and HIV treatments.

Symptoms of mental illness were common among our participants, but only one had a primary diagnosis of a mental health disorder. Failure to differentiate mental health disorders from meningitis can lead to delays in the diagnosis of meningitis. After a week's stay, with no improvement and worsening symptoms, a confirmed meningitis diagnosis was finally made in one of our participants who was initially thought to have only a mental health disorder. This case mirrored what other studies reported: symptoms and diagnosis of a mental illness were subsequently revised to a diagnosis of meningitis during hospitalization (M. Kumar et al. 2024; Thienhaus and Khosla 1984; Goeb et al. 2007; Laher et al. 2018). No other patients in our study who expressed issues with overthinking, depression, or stigma had been screened for mental illness or referred to a mental health professional during their hospitalization. These lapses in screening and lack of mental health referral contribute to missed opportunities for early identification and treatment of mental health conditions.

A key facilitator in both HIV and meningitis care was the physical and social support that patients experienced. Some participants were physically supported to come to the hospital, as they were weakened or unconscious, and therefore relying on family and friends to take them to the hospital saved their lives. Support from family, friends, other patients, and medical staff were contributors to participants’ sense of purpose and well‐being. Studies have also shown that the positive effects of social support, such as support groups, peer groups, and counseling for people living with HIV, improve adherence to ART and linkage to HIV care (Seffren et al. 2018; Lamb et al. 2014; Sanga et al. 2019). Having a support system, which provides encouragement to seek treatment, adhere to ART, and positive reinforcement, offers hope, motivation, and a renewed sense of purpose. Our findings highlight that physical, emotional, and spiritual support plays a crucial role in fostering hope and resilience and improves care. Therefore, recognizing the value of this type of support is essential and should not be overlooked.

### 4.1 Limitations

There are important limitations to this study. First, the use of phone calls excluded potential participants who did not have a recorded contact number. Lack of access to a phone may have excluded participants who had differing perceptions and experiences than our sample. Second, recall bias may have played a role among participants who were treated for meningitis 2–3 years ago, compared to participants who were seen within the past few months. In addition, family members likely had a harder time recalling certain details compared to patients, and the perceptions of family members may differ from the actual experiences of patients. To mitigate this limitation, when lapses in memory existed or conflicting information was given, these data were either classified as unknown or cross‐checked with clinical record forms to validate the information. Reporting bias may have influenced patients disproportionately when revealing sensitive topics of experiences with stigma, depression, or shame, as family members may have been more open to discuss these experiences compared to patients. Also, our study design utilized the point of data saturation in the qualitative component of our mixed‐methods approach to determine the sample size, and we utilized the same sample for the quantitative component. This approach allowed us to triangulate specific themes and measures across our patient population to facilitate the integration of qualitative and quantitative components. We acknowledge that this approach limits statistical inference and generalizability of the quantitative data from this exploratory study.

There were also strengths to this study, which revealed the lived experiences of patients with HIV, meningitis, and mental illness while capturing the nuanced personal and social contexts around HIV stigma. Incorporating family members as proxies for deceased patients provided unique insights into the patient's experience with meningitis, stigma, and mental illness symptoms and their broader ramifications, which would have otherwise remained unexplored.

## 5 Conclusion

Few participants were formally diagnosed with mental health disorders prior to our study, despite reported symptoms of mental illness. This finding highlights a gap in care regarding mental health screening among PLWH and meningitis patients within this rural hospital setting in northern Uganda. When patients present to hospitals with possible symptoms of mental illness or meningitis symptoms, they should be screened for mental illness during their meningitis evaluation to ensure proper care and reduce missed diagnoses. With integrated screening, we can further understand the prevalence of meningitis and mental health disorders in rural, northern Uganda.

Ongoing community‐based HIV education is essential to dispel misconceptions and reduce the stigma that persists among PLWH and their community. These misconceptions exacerbate stigma and perpetuate false narratives about HIV, leading to further isolation and shame. Incorporating targeted education and awareness campaigns can foster a more informed and supportive environment that is essential for improved well‐being. These strategies could mitigate stigma to improve treatment adherence and reduce the risks of severe mental illness among meningitis patients living with HIV.

## Author Contributions

**Abigail Link**: conceptualization, investigation, funding acquisition, writing – original draft, methodology, validation, visualization, writing – review and editing, formal analysis, project administration, data curation, supervision, resources, software. **Sarah Iribarren**: Metholology, writing – original draft, writing – review and editing. **Paul Bohjanen**: Funding acquisition, writing – original draft, writing – review and editing, project administration, resources, supervision. **Mark Okwir**: Writing – review and editing, project administration, supervision. **David Meya**: writing – original draft, writing – review and editing. **Betty Nabongo**: Project administration, writing – review and editing. **Danuta Kasprzyk**: conception, methodology, validation, visualization, writing – original draft, writing – review and editing, formal analysis, supervision.

## Ethics Statement

This study was approved by Gulu University Research Ethics Committee (GUREC 107–19), the Uganda National Council of Science and Technology (SS 5151), the University of Washington Institutional Review Board (IRB) (STUDY00007770), and the University of Minnesota IRB (STUDY00013187). Informed written or electronic consent was obtained from all participants before enrollment. For minors, written informed parental consent was obtained as well as written informed assents from adolescents.

## Data Availability

The data that support the findings of this study are available on request from the corresponding author.

## References

[brb371233-bib-0001] Akena, D. , S. Musisi , J. Joska , and D. J. Stein . 2012. “The Association Between Aids Related Stigma and Major Depressive Disorder Among HIV‐Positive Individuals in Uganda.” PLoS One 7, no. 11: e48671. 23209556 10.1371/journal.pone.0048671 PMC3507871

[brb371233-bib-0002] Bingham, A. J 2023. “From Data Management to Actionable Findings: A Five‐Phase Process of Qualitative Data Analysis.” International Journal of Qualitative Methods 22: 16094069231183620.

[brb371233-bib-0003] Breet, E. , A. Kagee , and S. Seedat . 2014. “HIV‐Related Stigma and Symptoms of Post‐Traumatic Stress Disorder and Depression in HIV‐Infected Individuals: Does Social Support Play a Mediating or Moderating Role?” Aids Care 26, no. 8: 947–951. 24666226 10.1080/09540121.2014.901486

[brb371233-bib-0004] Chibanda, D. , R. Verhey , L. J. Gibson , et al. 2016. “Validation of Screening Tools for Depression and Anxiety Disorders in a Primary Care Population With High HIV Prevalence in Zimbabwe.” Journal of Affective Disorders 198: 50–55. 27011359 10.1016/j.jad.2016.03.006

[brb371233-bib-0005] Elo, S. , and H. Kyngäs . 2008. “The Qualitative Content Analysis Process.” Journal of Advanced Nursing 62, no. 1: 107–115. 18352969 10.1111/j.1365-2648.2007.04569.x

[brb371233-bib-0006] England Public Health . 2019. “Trends in new HIV diagnoses and in People Receiving HIV‐Related Care in the United Kingdom: Data to the end of December 2018.” Health Protection Report 13, no. 31: 1–8.

[brb371233-bib-0007] Goeb, J. L. , V. Leon , and G. Kechid . 2007. “Cryptococcal Meningitis With Acute Psychotic Confusion in a Sarcoid Patient.” Primary Care Companion to the Journal of Clinical Psychiatry 9, no. 5: 393–394. 10.4088/pcc.v09n0511b PMC2040290 17998962

[brb371233-bib-0008] Haas, A. D. , C. Kunzekwenyika , S. Hossmann , et al. 2021. “Symptoms of Common Mental Disorders and Adherence to Antiretroviral Therapy Among Adults Living With HIV in Rural Zimbabwe: A Cross‐Sectional Study.” BMJ Open 11, no. 7: e049824. 10.1136/bmjopen-2021-049824 PMC8264908 34233999

[brb371233-bib-0009] Johansson Kostenniemi, U. , A. Bazan , L. Karlsson , and S. A. Silfverdal . 2021. “Psychiatric Disabilities and Other Long‐Term Consequences of Childhood Bacterial Meningitis.” Pediatric Infectious Disease Journal 40, no. 1: 26–31. 33021593 10.1097/INF.0000000000002908

[brb371233-bib-0010] Kumar, A. , S. Gopinath , K. R. Dinesh , and S. Karim . 2011. “Infectious Psychosis: Cryptococcal Meningitis Presenting as a Neuropsychiatry Disorder.” Neurology India 59, no. 6: 909–911. 22234213 10.4103/0028-3886.91379

[brb371233-bib-0011] Kumar, M. , H. Nigma , C. Rajarshi , K. Wangda , and I. Khan . 2024. “Psychosis in Tuberculous Meningitis: A Case Series.” Journal of Clinical Case Studies 13, no. 2: 62–64.

[brb371233-bib-0012] Laher, A. E. , Y. Etlouba , M. Moolla , F. Motara , and N. Ariefdien . 2018. “First‐Presentation With Psychotic Behavior to the Emergency Department: Meningitis or Not, That Is the Question.” American Journal of Emergency Medicine 36, no. 11: 2068–2075. 30190242 10.1016/j.ajem.2018.08.057

[brb371233-bib-0013] Lamb, M. R. , R. Fayorsey , H. Nuwagaba‐Biribonwoha , et al. 2014. “High Attrition Before and After ART Initiation Among Youth (15–24 Years of age) Enrolled in HIV Care.” Aids 28, no. 4: 559–568. 24076661 10.1097/QAD.0000000000000054 PMC4517438

[brb371233-bib-0014] Lundberg, P. , N. Nakasujja , M. Seggane , A. Thorson , E. Cantor‐Graae , and P. Allebeck . 2013. “HIV Prevalence in Persons With Severe Mental Illness in Uganda: A Cross‐Sectional Hospital‐Based Study.” International Journal of Mental Health Systems 7: 20. 23866085 10.1186/1752-4458-7-20 PMC3724693

[brb371233-bib-0015] Molodynski, A. , C. Cusack , and J. Nixon . 2017. “Mental Healthcare in Uganda: Desperate Challenges but Real Opportunities.” BJPsych International 14, no. 4: 98–100. 29093962 10.1192/s2056474000002129 PMC5663025

[brb371233-bib-0016] Montaño, D. E. , and D. Kasprzyk . 2015. “Theory of Reasoned Action, Theory of Planned Behavior, and the Integrated Behavioral model.” In Health Behavior: Theory, Research, and Practice, edited by K. Glanz , B. K. Rimer , and K. Viswanath , 95–124. Jossey‐Bass/Wiley.

[brb371233-bib-0017] Mugisha, J. , C. Hanlon , B. L. Knizek , et al. 2019. “The Experience of Mental Health Service Users in Health System Strengthening: Lessons From Uganda.” International Journal of Mental Health Systems 13: 60. 31516548 10.1186/s13033-019-0316-5 PMC6728966

[brb371233-bib-0018] Mugisha, J. , H. Muyinda , P. Wandiembe , and E. Kinyanda . 2015. “Prevalence and Factors Associated With Posttraumatic Stress Disorder Seven Years After the Conflict in Three Districts in northern Uganda (The Wayo‐Nero Study).” BMC Psychiatry 15: 170. 26205192 10.1186/s12888-015-0551-5 PMC4513792

[brb371233-bib-0019] Mutegeki, G 2019. Millions of Ugandans Suffering from Depression Unknowingly Kampala, Uganda: New Vision . https://www.newvision.co.ug/new_vision/news/1495716/millions‐ugandans‐suffering‐depression‐unknowingly.

[brb371233-bib-0020] Patel, V. , E. Simunyu , F. Gwanzura , G. Lewis , and A. Mann . 1997. “The Shona Symptom Questionnaire: The Development of an Indigenous Measure of Common Mental Disorders in Harare.” Acta Psychiatrica Scandinavica 95, no. 6: 469–475. 9242841 10.1111/j.1600-0447.1997.tb10134.x

[brb371233-bib-0021] R Core Team . 2020. R: A Language and Environment for Statistical Computing. 3.6.3 ed. R Foundation for Statisical Computing.

[brb371233-bib-0022] Rahim, M. J. , and W. S Ghazali . 2016. “Psychosis Secondary to Tuberculosis Meningitis.” BMJ Case Reports 2016: bcr2015213171. 10.1136/bcr-2015-213171 PMC4800240 26969352

[brb371233-bib-0023] Rajasingham, R. , D. B. Meya , G. S. Greene , et al. 2019. “Evaluation of a National Cryptococcal Antigen Screening Program for HIV‐Infected Patients in Uganda: A Cost‐Effectiveness Modeling Analysis.” PLoS One 14, no. 1: e0210105. 30629619 10.1371/journal.pone.0210105 PMC6328136

[brb371233-bib-0024] Rezaei, S. , S. Ahmadi , J. Rahmati , et al. 2019. “Global Prevalence of Depression in HIV/AIDS: A Systematic Review and Meta‐Analysis.” BMJ Supportive & Palliative Care 9, no. 4: 404–412. 10.1136/bmjspcare-2019-001952 31537580

[brb371233-bib-0025] Roberts, B. , K. F. Ocaka , J. Browne , T. Oyok , and E. Sondorp . 2008. “Factors Associated With Post‐Traumatic Stress Disorder and Depression amongst Internally Displaced Persons in northern Uganda.” BMC Psychiatry 8, no. 1: 38. 18489768 10.1186/1471-244X-8-38 PMC2397420

[brb371233-bib-0026] Rueda, S. , S. Mitra , S. Chen , et al. 2016. “Examining the Associations Between HIV‐Related Stigma and Health Outcomes in People Living With HIV/AIDS: A Series of Meta‐Analyses.” BMJ Open 6, no. 7: e011453. 10.1136/bmjopen-2016-011453 PMC4947735 27412106

[brb371233-bib-0027] Ruffell, S. 2017. “Stigma Kills! the Psychological Effects of Emotional Abuse and Discrimination Towards a Patient With HIV in Uganda.” BMJ Case Reports 2017: bcr2016218024. 10.1136/bcr-2016-218024 PMC5534769 28710190

[brb371233-bib-0028] Sandler, N. H 1975. “A Case of Meningitis Admitted as Schizophrenia.” Journal of the Kentucky Medical Association 73, no. 1: 25–26. 1113021

[brb371233-bib-0029] Sanga, E. S. , F. C. Mukumbang , A. K. Mushi , W. Lerebo , and C. Zarowsky . 2019. “Understanding Factors Influencing Linkage to HIV Care in a Rural Setting, Mbeya, Tanzania: Qualitative Findings of a Mixed Methods Study.” BMC Public Health 19, no. 1: 383. 30953503 10.1186/s12889-019-6691-7 PMC6451278

[brb371233-bib-0030] Sayles, J. N. , M. D. Wong , J. J. Kinsler , D. Martins , and W. E. Cunningham . 2009. “The Association of Stigma With Self‐Reported Access to Medical Care and Antiretroviral Therapy Adherence in Persons Living With HIV/AIDS.” Journal of General Internal Medicine 24, no. 10: 1101–1108. 19653047 10.1007/s11606-009-1068-8 PMC2762503

[brb371233-bib-0031] Seffren, V. , I. Familiar , S. M. Murray , et al. 2018. “Association Between Coping Strategies, Social Support, and Depression and Anxiety Symptoms Among Rural Ugandan Women Living With HIV/AIDS.” Aids Care 30, no. 7: 888–895. 29471677 10.1080/09540121.2018.1441969 PMC9850497

[brb371233-bib-0032] Sessions, K. L. , L. Wheeler , A. Shah , et al. 2017. “Mental Illness in Bwindi, Uganda: Understanding Stakeholder Perceptions of Benefits and Barriers to Developing a Community‐Based Mental Health Programme.” African Journal of Primary Health Care & Family Medicine 9, no. 1: e1–e7. 10.4102/phcfm.v9i1.1462 PMC5803513 29227132

[brb371233-bib-0033] Shumba, C. , R. Atukunda , R. Imakit , and P. Memiah . 2013. “Prevalence of Depressive Symptoms Amongst Highly Active Antiretroviral Therapy (HAART) Patients in AIDSRelief Uganda.” Journal of Public Health in Africa 4, no. 2: e19. 28299108 10.4081/jphia.2013.e19 PMC5345437

[brb371233-bib-0034] Thienhaus, O. J. , and N. Khosla . 1984. “Meningeal Cryptococcosis Misdiagnosed as a Manic Episode.” American Journal of Psychiatry 141, no. 11: 1459–1460. 6496792 10.1176/ajp.141.11.1459

[brb371233-bib-0035] Titeca, K. C. , and T. Costeur . 2014. “An LRA for Everyone: How Different Actors Frame the Lord's Resistance Army.” African Affair 454, no. 114: 92–114.

[brb371233-bib-0036] Uganda Ministry of Health (MOH) . 2012. Uganda Aids Indicator Survey 2011. MOH.

[brb371233-bib-0037] Uganda Ministry of Health . 2022. Uganda Population‐Based HIV Impact Assessment. Uganda Ministry of Health.

[brb371233-bib-0038] Uganda Ministry of Health . 2023. Uganda Clinical Guidelines 2023. Uganda Ministry of Health.

[brb371233-bib-0039] Ursoiu, F. , L. Moleriu , D. Lungeanu , and M. Puschita . 2018. “The Association Between HIV Clinical Disease Severity and Psychiatric Disorders as Seen in Western Romania.” Aids Care 30, no. 11: 1368–1371. 29592527 10.1080/09540121.2018.1455959

[brb371233-bib-0040] US Department of Health and Human Services . 2019. HIV and Mental Health. National Institutes of Health (NIH).

[brb371233-bib-0041] Wilkinson, R. J. , U. Rohlwink , U. K. Misra , et al. 2017. “Tuberculous Meningitis.” Nature Reviews Neurology 13, no. 10: 581–598. 28884751 10.1038/nrneurol.2017.120

[brb371233-bib-0042] World Health Organisation (WHO) . 2011. Global HIV/AIDS Response: Epidemic Update and Health Sector Progress Towards Universal Access: Progress Report 2011'. WHO.

[brb371233-bib-0043] World Health Organisation (WHO) . 2023. Meningitis Geneva. WHO. https://www.who.int/news‐room/fact‐sheets/detail/meningitis.

[brb371233-bib-0044] World Health Organization (WHO) . 2015. Managing Meningitis Epidemics in Africa: A Quick Reference Guide for Health Authorities and Health‐Care Workers. World Health Organization. 26110196

[brb371233-bib-0045] World Health Organization (WHO) . 2016. “Meningitis Control in Countries of the African Meningitis Belt, 2015.” Weekly Epidemiological Record 91, no. 16: 209–216. 27108455

[brb371233-bib-0046] World Health Organization (WHO) . 2017. Primary Health Care Systems (PRIMASYS): Case Study From Uganda, Abridged Veresion. World Health Organization.

[brb371233-bib-0047] World Health Organization (WHO) . 2020. Defeating Meningitis by 2030: A Global Road Map. World Health Organization.

[brb371233-bib-0048] Wunrow, H. Y. , R. G. Bender , A. Vongpradith , et al. 2023. “Global, Regional, and National Burden of Meningitis and Its Aetiologies, 1990–2019: A Systematic Analysis for the Global Burden of Disease Study 2019.” Lancet Neurology 22, no. 8: 685–711. 37479374 10.1016/S1474-4422(23)00195-3 PMC10356620

